# Safety, effectiveness, and economic evaluation of an herbal medicine, Ukgansangajinpibanha granule, in children with autism spectrum disorder: a study protocol for a prospective, multicenter, randomized, double-blinded, placebo-controlled, parallel-group clinical trial

**DOI:** 10.1186/s13063-019-3537-7

**Published:** 2019-07-15

**Authors:** Sun Haeng Lee, Seungwon Shin, Tae-hun Kim, Sang Min Kim, Tae Yoon Do, Sulgi Park, Boram Lee, Hye Jin Shin, Jihong Lee, Jin Yong Lee, Gyu Tae Chang

**Affiliations:** 1 Department of Clinical Korean Medicine, Graduate School, Kyung Hee University, 26 Kyungheedae-ro, Dongdaemun-gu, Seoul, 02447 Republic of Korea; 2 Department of Pediatrics of Korean Medicine, Kyung Hee University Korean Medicine Hospital, Kyung Hee University Medical Center, 23 Kyungheedae-ro, Dongdaemun-gu, Seoul, 02447 Republic of Korea; 3 Department of Korean Medicine, Kyung Hee University, 26 Kyungheedae-ro, Dongdaemun-gu, Seoul, 02447 Republic of Korea; 4 Korean Medicine Clinical Trial Center, Kyung Hee University Korean Medicine Hospital, Kyung Hee University Medical Center, 23 Kyungheedae-ro, Dongdaemun-gu, Seoul, 02447 Republic of Korea; 5 Department of Pediatrics of Korean Medicine, Kyung Hee University Hospital at Gangdong, 892 Dongnam-ro, Gangdong-gu, Seoul, 05278 Republic of Korea

**Keywords:** Herbal medicine, Acupuncture, Autism spectrum disorder, Randomized controlled trial, Ukgansangajinpibanha

## Abstract

**Background:**

Autism spectrum disorder (ASD) is characterized by continuous impairment in communication and social interaction and by limited and repetitive behaviors, interests, or activities. Behavioral, educational, and pharmaceutical interventions have been shown to reduce behavioral disabilities, improve verbal/non-verbal communication, and help patients acquire self-reliance skills. However, there has been a lack of systematic verification and consensus regarding the treatment of the core symptoms of ASD because of its unclear etiology. Ukgansangajinpibanha (UGSJB), a legitimately prescribed herbal medicine for nervousness, insomnia, night crying, and malnutrition in South Korea and Japan, has been used for angry, sensitive, nervous, and unsettled children with ASD.

**Methods/design:**

This trial is a prospective, multicenter, randomized, double-blinded, placebo-controlled, parallel-group, clinical trial. The 4- to 6-year-old children with ASD will be randomly assigned to following groups:

1. A UGSJB granule with acupuncture, twice daily (n = 120)
2. A placebo group with acupuncture, twice daily (n = 120).

The following outcome measures will be used: behavior by the Childhood Autism Rating Scale, Autism Behavior Checklist, and Aberrant Behavior Checklist; social maturity by the Social Maturity Scale; quality of life by the Child Health Questionnaire and EuroQoL Five-dimension Five-level Youth; and parental stress by the Parenting Stress Index at baseline and at 6, 12, and 24 weeks after the beginning of treatment.

In addition, to evaluate safety, we will investigate the adverse reactions that may be caused by UGSJB granule. Finally, we will make an economic evaluation of UGSJB for the treatment of ASD.

**Discussion:**

We prepared a well-designed clinical trial to investigate the safety and effectiveness of UGSJB on ASD symptoms compared with placebo treatment. The results from this study will provide clinical evidence on the safety, effectiveness, and economic value of UGSJB combined with acupuncture in children with ASD.

**Trial registration:**

Clinical Research Information Service: KCT0003007 (registered on April 5, 2018)

**Electronic supplementary material:**

The online version of this article (10.1186/s13063-019-3537-7) contains supplementary material, which is available to authorized users.

## Background

Autism spectrum disorder (ASD) is characterized by continuous impairment in communication and social interaction (diagnostic criteria A) and by limited and repetitive behaviors, interests, or activities (diagnostic criteria B). These symptoms appear early in childhood and negatively affect daily functions (diagnostic criteria C and D) according to the *Diagnostic and Statistical Manual of Mental Disorders* (5th edition) [1]. The prevalence rate of ASD is four times higher for boys than for girls and has increased since the 1970s, especially from the late 1990s [2]. The prevalences of ASD are 2–20 per 1000 people in recent studies in Europe, Asia, and the US and 7.6 per 1000 people according to a global systematic review in 2010 [3]. Causes of ASD are unclear, but gene abnormalities altering brain development, brain damage at birth, and maternal effects during pregnancy have been suggested.

General therapeutic goals of ASD are to reduce behavioral disabilities, improve verbal/non-verbal communication, and provide patients with self-reliance skills. Non-pharmaceutical treatments aim to improve ASD symptoms through individualized behavioral and educational interventions, including behavioral and developmental programs, cognitive behavioral therapies, occupational therapies, sensory integration therapies, sleep management, and communication facilitation [4]. Pharmaceutical treatments, including risperidone, aripiprazole, methylphenidate, and haloperidol, are generally used as adjunctive therapies to reduce the accompanying symptoms such as aggression, anxiety, depression, hyperactivity, stereotypic and self-harm behavior, and insomnia rather than the core symptoms of ASD [5]. However, there has been a lack of systematic verification and consensus regarding the treatment of the core symptoms of ASD because of its unclear etiology.

ASD children can be hurt psychologically by passively accepting many criticisms in life because of their unusual behavior. In traditional Chinese medicine (TCM) theory, the persistent emotional stimulus affects the function of liver free coursing and causes stagnation of liver qi. Dysfunction of liver free coursing is often found in the early stages of ASD, which are characterized by mental depression and apathetic expression. Depressed liver qi transforms into fire, which is characterized by agitation and anger, red face and eyes, and constipation and yellow urine. Prolonged stagnation of liver qi causes growth retardation, introverted behavior, and an autistic state. ASD children disregard other people, fail to look at each other, and avoid one’s eyes. Those are also dysfunctions of liver free coursing because, from a TCM perspective, liver function is reflected in the activity of the eyes.

Ukgansan (抑肝散 in Chinese, Yokukansan in Japanese), an herbal medicine consisting of Atractylodis Rhizoma White, Poria, Angelicae Gigantis Radix, Cnidii Rhizoma, Uncariae Ramulus et Uncus, Bupleuri Radix, and Glycyrrhizae Radix, was made by Xue Ji in 1556 for treating children’s liver dysfunction such as contracture, convulsion, and epilepsy [6]. It inhibits hypersensitivity and hyperactivity of ASD through anti-inflammatory actions, neurogenesis, and serotonin and glutamate upregulation [7]. In a neurodevelopmental disorder model, Ukgansan decreases grooming in the open field [8]. Ukgansan and Gyejitang (桂枝湯) prevent behavioral abnormalities and improve neuroplasticity signaling in a social isolation model in mice [9]. In a 12-year-old boy with ASD, Ukgansan improved irritability, which had not been successfully treated with anti-psychotic drugs [10]. Ukgansan was administered to 20 children (6–17 years old) with pervasive developmental disorder (PDD) for 12 weeks; as a result, irritability/agitation and hyperactivity/non-compliance subscale scores of the Aberrant Behavior Checklist (ABC^2^) were significantly improved [11]. In another study, Ukgansan was administered to 40 patients (8–40 years old) with PDD-not otherwise specified or Asperger’s syndrome for 12 weeks, and ABC^2^ irritability subscale scores were also significantly improved [12]. However, the effectiveness of Ukgansan or similar prescriptions has not yet been investigated in randomized controlled trials (RCTs).

Ukgansangajinpibanha (UGSJB), which is made from Ukgansan by adding Citri Pericarpium and Pinelliae Rhizoma, has been more often used in children with indigestion compared with Ukgansan. Similar to Ukgansan, it has been administered to angry, sensitive, nervous, and unsettled children with ASD [13]. Unlike Ukgansan, UGSJB is legitimately prescribed as an herbal formula granule for nervousness, insomnia, night crying, and malnutrition in South Korea and Japan [14]. Interestingly, children with ASD tend to experience more gastrointestinal symptoms than children without ASD [15]. Therefore, we prepared a well-designed clinical trial to investigate the safety and effectiveness of UGSJB on ASD symptoms compared with placebo treatment. The results of this trial will provide evidence of whether UGSJB is safe and effective for the treatment of ASD in children.

Acupuncture, a treatment supported by the National Health Service in South Korea, will be provided to every participant for an economic evaluation. Acupuncture has weak evidence of benefits but is a safe and effective treatment for ASD [16]. Frequently used acupoint for treating ASD sympstoms are EX-HN1, GV17, PC6, LR3, KI3, HT7, LU9, GV20, GV24, GV24.5, GB13, GB19, KI4, LR4, ST36, SP3, and SP6 [17]. We decided to use EX-HN1, GV20, and GV24 for acupuncture treatment of ASD in children because these acupoints have been frequently used in RCTs on ASD [18], and scalp acupuncture decreased scores of Childhood Autism Rating Scale (CARS) and Autism Behavior Checklist (ABC^1^) of children with ASD [19]. UGSJB in combination with acupuncture will be evaluated from an economic perspective.

## Methods/design

### Study design

This study is a prospective, multicenter, randomized, double-blinded, placebo-controlled, parallel-group, clinical trial. The aim of the clinical trial is to evaluate the effectiveness of UGSJB in 4- to 6-year-old children with ASD, including childhood autism, Asperger’s syndrome, and PDD-not otherwise specified, diagnosed according to the Korean Standard Classification of Diseases (7th edition) by any physician who has not participated in this study. The study protocol conforms to the Standard Protocol Items: Recommendations for Interventional Trials (Additional file 1), Consolidated Standards of Reporting Trials extension for reporting herbal medicines, and Standards for Reporting Interventions in Clinical Trials of Acupuncture. The trial will be performed in accordance with the Declaration of Helsinki and the Ethical Guideline for Clinical Research, and the trial protocol has been approved by the Ministry of Food and Drug Safety (#31635) and the institutional review board (IRB) at Kyung Hee University Korean Medicine Hospital at Gangdong (KHNMCOH 2017–08-008) and Kyung Hee University Korean Medicine Hospital (KOMCIRB 2017–10-041). The trial protocol has been registered with Clinical Research Information Service (KCT0003007) (Appendix: Table 2).

### Study participants

**Fig. 1:**
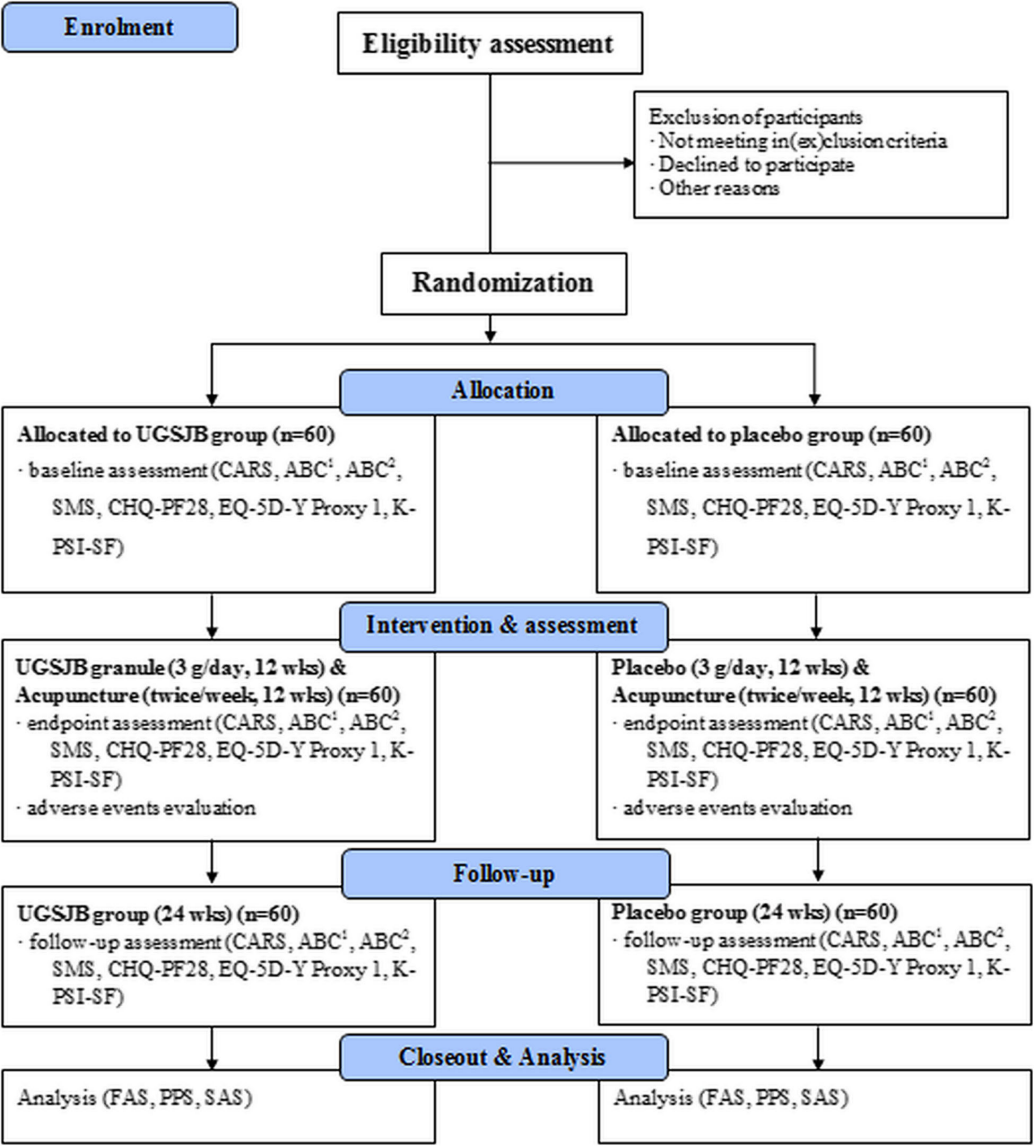
Schematic chart of the process of the clinical trial. *Abbreviations*: *ABC*^*1*^ Autism Behavior Checklist, *ABC*^*2*^ Aberrant Behavior Checklist, *ACER* Average cost-effectiveness ratio, *ACUR* Average cost-utility ratio, *AE* Adverse event, *ANCOVA* Analysis of covariance, *ASD* Autism spectrum disorder, *CARS* Childhood Autism Rating Scale, *CBA* Cost-benefit analysis, *CEA* Cost-effectiveness analysis, *CHQ-PF28* Child Health Questionnaire-Parent Report Form 28, *CUA* Cost-utility analysis, *EQ-5D-Y* EuroQoL Five-dimension Five-level Youth, *FAS* Full analysis set, *ICER* Incremental cost-effectiveness ratio, *IRB* Institutional review board, *K-CTC* Korean Medicine Clinical Trial Center, *K-PSI-SF* Korean Parenting Stress Index Short Form, *PDD* Pervasive developmental disorder, *PPS* Per protocol set, *PSI* Parenting Stress Index, *RCT* Randomized controlled trial, *SAS* Safety Assessment Set, SMS Social Maturity Scale, *TCM* Traditional Chinese Medicine,*UGSJB* Ukgansangajinpibanha

**Fig. 2:**
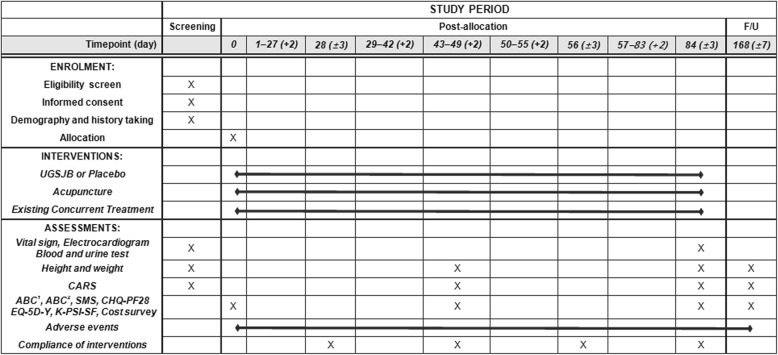
Schedule of enrolment, interventions, and assessments. *Abbreviations*: *ABC*^*1*^ Autism Behavior Checklist, *ABC*^*2*^ Aberrant Behavior Checklist, *CARS* Childhood Autism Rating Scale, *CHQ-PF28* Child Health Questionnaire-Parent Report Form 28, *EQ-5D-Y* EuroQoL Five-dimension Five-level Youth, *K-PSI-SF* Korean Parenting Stress Index Short Form, *SMS* Social Maturity Scale, *UGSJB* Ukgansangajinpibanha

One hundred twenty children will be recruited from two hospitals (Kyung Hee University Hospital at Gangdong and Kyung Hee University Medical Center, Republic of Korea) through online and offline recruitment notices, including posters and webpages. Those two hospitals have recruited participants in more than 20 clinical trials that eight studies were completed and 19 studies are recruiting in Kyung Hee University Hospital at Gangdong, and 41 studies were completed and 52 studies are recruiting in Kyung Hee University Medical Center. The participants’ guardians will be provided with research information, such as objectives, procedures, and potential benefits and harms, through standardized interviews. Informed consent will be obtained from children’s guardians prior to the screening process. The participants will be allowed to withdraw from the study at any time. The research procedure will be carried out as shown in Figs. 1 and 2. All participants will undergo a 12-week treatment and 12-week follow-up. After the screening session, children will be scheduled for 24 treatment visits (twice a week) and one follow-up visit (12 weeks after last treatment). Guardians will be sent text messages as reminders of the scheduled visits.

### Inclusion criteria

1. Children who are 4 to 6 years old
2. Children who have received an ASD-specific medical certificate from their physician (e.g., autistic disorder, Asperger’s syndrome, or PDD-not otherwise specified)
3. CARS score of at least 30 in the screening test
4. Written informed consent signed by a representative.

### Exclusion criteria

1. Hereditary syndromes, including Rett syndrome, Down syndrome, or fragile X syndrome
2. Other mental disorders, including selective mutism, attention-deficit/hyperactivity disorder, schizophrenia, or depressive disorder
3. Hereditary metabolic diseases, including galactose intolerance, Lapp lactase deficiency, or glucose-galactose malabsorption
4. Recently operated children, seizures (including epilepsy), generalized infections requiring antibiotics, brain injuries (including cerebral palsy), or severe chronic or terminal illness (including malignancy or tuberculosis)
5. Children who have taken risperidone or aripiprazole or who have stopped taking these medications less than 3 months prior to enrolment in the study
6. Children who have taken any Korean medicine in the past month prior to enrolment in the study
7. Aspartate aminotransferase, alanine aminotransferase, blood urea nitrogen, or creatinine level more than three times the normal upper limit
8. Hypersensitivity to the test drug or placebo
9. Difficulty in swallowing the test drug or placebo
10. Intolerance or fear of acupuncture
11. Hemostatic disorders
12. Children who had participated in another clinical trial within 1 month of the enrolment date
13. Inability to participate in the trial as determined by the trial director or personnel.

### Interventions

Already-prescribed treatments—including medications such as methylphenidate, haloperidol, and selective serotonin reuptake inhibitors; behavioral and educational interventions such as communication interventions, social skills instruction, and occupational therapy; and complementary and alternative therapies such as music therapy, animal therapy, and transcranial magnetic stimulation—will be continued during the trial. Stopping existing treatments and starting new treatments will be restricted during the trial. The use of risperidone, aripiprazole, and any herbal medicine except UGSJB or placebo will be forbidden. Potassium agents, licorice agents, glycyrrhizinate agents, and diuretic agents which can cause myopathy by pseudoaldosteronism or hypokalemia will be used cautiously only if needed.

After screening, suitable participants will be randomly assigned to the 3.0 g/day UGSJB or placebo group. UGSJB granule is composed of nine herbs: Ban Xia (*Pinellia ternate*), Bai Zhu (*Atractylodes japonica*), Fu Ling (*Poria cocos*), Dang Gui (*Angelica gigas*), Chen Pi (*Citrus unshiu*), Chuan Xiong (*Cnidium officinale*), Diao Gou Teng (*Uncaria sinensis*), Chai Hu (*Bupleurum falcatum*), and Gan Cao (*Glycyrrhiza glabra*) (Table 1). Placebo granule is composed of 0.95 g lactose, 0.4 g corn starch, and 0.15 g caramel pigment to match the UGSJB. UGSJB and placebo granules were manufactured and stored by Kyungjin Pharmaceutical Co., Ltd. (Gyeonggi, Republic of Korea). The solvent used in the extract was purified water, and the ratio of extract to herbal drugs was about 12.2%. Storage, dispensation, and quality control of the UGSJB and placebo granules were carried out by a blinded pharmacist in each study center. Caregivers will be instructed to dissolve in water and administer one pack (1.5 g) of UGSJB or placebo granule after breakfast and dinner for 12 weeks, which was the period that reported effectiveness in previous studies [11, 12]. The dosage regimen was determined by referring to the authorization by the Ministry of Food and Drug Safety and the clinical judgment of Korean medicine doctors. The quantities of marker constituents per pack were 3.99 mg of glycyrrhizinate and 11.18 mg of hesperidin. Lead and microorganisms were not detected, and the concentration of arsenic was 0.33 parts per million in one pack of UGSJB. The UGSJB or placebo will be prescribed by Korean medicine doctors with more than 5 years of clinical experience. If there are any adverse events (AEs) related to UGSJB, such as pseudoaldosteronism, myopathy, allergic skin reaction, and gastrointestinal upset, that participant will stop taking medication and will receive appropriate treatment according to the severity of the AEs. The guardians should return the used as well as unopened packages to check the adherence to treatment.

**Table 1:** Composition of Ukgansangajinpibanha granule

Plant name	Used part	Registration	Amount, grams
*Pinellia ternate* Breitenbach (Araceae)	Dried rhizome	KP	0.84
*Atractylodes japonica* Koidz. (Compositae)	Dried rhizome	KP	0.67
*Poria cocos* Wolf (Polyporaceae)	Dried scleorotia	KP	0.67
*Angelica gigas* Nakai (Umbelliferae)	Dried root	KP	0.5
*Citrus unshiu* Markovich (Rutaceae; citrus peel)	Dried fruit peel	KP	0.5
*Cnidium officinale* Makino (Umbelliferae)	Dried rhizome	KP	0.5
*Uncaria sinensis* Havil. (Rubiaceae)	Dried ramulus	KHP	0.5
*Bupleurum falcatum* Linné (Umbelliferae)	Dried root	KP	0.34
*Glycyrrhiza glabra* L. (Leguminosae; licorice)	Dried root and rhizome	KP	0.25

*Abbreviations*: *KHP* Korean Herbal Pharmacopoeia, *KP* Korean Pharmacopoeia.

Scalp acupuncture will be performed in all participants by using six fixed points at GV20 (Bai Hui), GV24 (Shen Ting), and EX-HN1 (Si Shen Cong) in a total of 24 sessions for 12 weeks. We considered participants who attended 75% or more (≥18 of 24) of the acupuncture sessions to have completed a full course of treatment. A 0.25 × 40 mm disposable sterilized stainless-steel needle will be used. GV20 and GV24 were inserted backward and EX-HN1 was inserted outward. After insertion of the needle (10–15 mm of depth), it will be stirred by hand until de qi sensation. The inserted needle will remain for 30 min with available manual stimulation. If the child pulls out the needles during the procedure, other needles will be inserted. Acupuncture will be performed by Korean medicine doctors with 3 years of acupuncture training and more than 1 year of clinical experience. If the participant strongly complains of discomfort, the practitioner will stop the acupuncture procedure.

### Random assignment

Subjects who have agreed to participate in this clinical trial are assigned a screening number in the order of their guardian’s written informed consent. After completing the screening test, eligible subjects will be assigned to the UGSJB or placebo group according to their random assignment code. The random assignment codes are provided by an independent statistician in the Korean Medicine Clinical Trial Center (K-CTC), and random sequence numbers were generated by blockrand package 1.3 of R 3.4.2 (2017-09-28). The block random assignment will be made after stratification according to the hospital so that the subjects of each group are approximately identical.

#### Allocation concealment

The independent statistician who generated the random assignment code or a central researcher in K-CTC who does not have contact with the subjects provided the random assignment table directly to Kyungjin Pharmaceutical Co., Ltd., and the company created a label for the medications to be used in the clinical trial. The random assignment code will be kept only by the independent statistician, the central researcher, and the pharmaceutical company personnel during the clinical trial period and will not be disclosed to any subject unless justified by a pre-defined reason.

#### Blinding

Subjects, acupuncturists, data collectors, and statistical analysts will be blinded to which medication will be administered to subjects by using placebo as a control. The placebo will have the same formulation and properties of the UGSJB granule to prevent any bias in safety and efficacy assessments. Unblinding should be considered on a case-by-case basis and only if severe medical emergencies occur or administration information affects the treatment of the subjects.

### Primary outcome measures

#### Childhood Autism Rating Scale

CARS was developed to distinguish autism from other developmental disorders and to assess the severity of autism. It has high reliability, validity, and inter-rater consistency, and a cutoff score of 30 points distinguished autism from other developmental disorders. In addition, CARS reported a high rate of consistency of 92% with experts when used after short training, even if the rater has little experience with autism. In the Korean version of CARS, the internal consistency coefficient (Cronbach’s α) is 0.87, the inter-rater agreement is 0.94, and the test-retest reliability is 0.91 [20]. A total score of 30–37 indicates mild autism, and a total score of 37.5–60 indicates severe autism. In this clinical trial, the assessment will be carried out during the visit at baseline (visit 1), mid-point (visit 14), termination (visit 25), and follow-up (visit 26).

### Secondary outcome measures

#### Autism Behavior Checklist (ABC^1^)

ABC^1^ was developed by Krug *et al*. for differentiating autistic children from intellectual disability, deaf-blind, emotionally disturbed, or normal children [21]. It provides insight into the general characteristics of what an individual looks like compared with another person. ABC^1^ has a reported internal reliability of 0.87 [22], sensitivity of 0.38–0.58, specificity of 0.76–0.97 [23], and test-retest reliability of 0.83 [24]. A child with a total score above 67 is highly likely to be autistic, and those with a total score between 53 and 67 require further evaluation. The assessment will be carried out during the visit at baseline (visit 2), mid-point (visit 14), termination (visit 25), and follow-up (visit 26).

#### Aberrant Behavior Checklist (ABC^2^)

ABC^2^ was developed by Aman *et al*. to measure the treatment effectiveness for people with intellectual disabilities [25] and has been widely used for measuring the progress of treatment for PDD [26]. Cronbach’s α ranges from 0.86 to 0.94, and the inter-rater reliability is 0.63 [27]. Higher scores mean a greater degree of aberrant behavior. The assessment will be carried out during the visit at baseline (visit 2), mid-point (visit 14), termination (visit 25), and follow-up (visit 26).

#### Social Maturity Scale

The Social Maturity Scale (SMS) was designed to assess a person’s level of development by identifying the actual needs and responsibilities of an individual in daily life. Questions are presented depending on age, and social age is calculated by interpolation of the total score [28]. Higher scores mean a greater degree of social maturity. The assessment will be carried out during the visit at baseline (visit 2), mid-point (visit 14), termination (visit 25), and follow-up (visit 26).

#### Child Health Questionnaire-Parent Report Form 28 (CHQ-PF28)

CHQ-PF28 has been used around the world as a tool to measure the general quality of life for children who are 5 or older. It has a verified internal consistency of 0.72 and a test-retest reliability of 0.31–0.84 [29]. The item scores are aggregated according to the calculation method set by HealthActCHQ, Inc. (Boston, MA, USA), and the higher score is interpreted as the higher quality of life. The assessment will be carried out during the visit of baseline (visit 2), mid-point (visit 14), termination (visit 25), and follow-up (visit 26).

#### EuroQoL Five-dimension Five-level Youth (EQ-5D-Y) proxy 1

EQ-5D-Y was transformed from EQ-5D to assess the quality of life of children and teens, and the proxy version is recommended for ages 4 to 7. The results of the multinational studies show that EQ-5D-Y has a test-retest reliability of 69.8–99.7%, Kappa coefficient of 0.67, and correlation coefficient of −0.56 with other tools [30]. Lower scores indicate better quality of life. The assessment will be carried out during the visit at baseline (visit 2), mid-point (visit 14), termination (visit 25), and follow-up (visit 26).

#### Korean Parenting Stress Index Short Form

Korean Parenting Stress Index Short Form (K-PSI-SF) was designed to measure the relative magnitude of stress that is experienced by parents in parent–child relationships. It is standardized for use on parents of children between the ages of 1 month and 12 years. For the US version of PSI, the internal consistency values were 0.70–0.83 in the children’s section, 0.70–0.84 in the parents’ section, and higher than 0.9 in the total stress score; the test-retest reliability values were 0.63 in the children’s section, 0.91 in the parents’ section, and 0.96 in the total stress score. For the Korean version of PSI, the Cronbach’s internal consistency was 0.76–0.91 and the test-retest reliability was 0.69–0.77 [31]. Scores of 15–80 percentiles are interpreted as normal range, 81–84 percentiles are boundary levels, and above 85 percentiles are risk levels. The assessment will be carried out during the visit at baseline (visit 2), mid-point (visit 14), termination (visit 25), and follow-up (visit 26).

### Safety assessment

To evaluate the safety of UGSJB, electrocardiogram and laboratory examinations (blood routine test, urine routine test, liver and kidney function test, and electrolytes) will be conducted on all participants at the screening phase (week 0) and post-treatment phase (week 12). At each visit, voluntarily reporting subjective or objective adverse symptoms will be collected to assess overall clinical safety by asking the question “Has there been any abnormal reactions since the last visit?” If participants drop out of our trial for treating any AEs, the AEs will be considered severe.

### Economic evaluation

Cost-effectiveness analysis (CEA), cost-utility analysis (CUA), and cost-benefit analysis (CBA) will be carried out for the economic evaluation. Cost variables include medical and non-medical costs of patients and cost burden and productivity losses of guardians. Effectiveness variables will be measured by ABC^2^, CARS, and CHQ-PF28. Utility variable will be measured by EQ-5D-Y proxy 1. The result of CEA will be calculated as the average cost-effectiveness ratio (ACER) or incremental cost-effectiveness ratio (ICER), and the result of CUA will be calculated as the average cost-utility ratio (ACUR). Intervention with the smallest ACER or ACUR will be selected. Intervention with ICER below a certain level will be considered cost-effective.

### Sample size calculation

In Zhou *et al*. (2015) [32], an RCT on autistic children (average age of 4.75 years) was conducted to measure the CARS before and after the interventions in the herbal medicine–plus–behavioral education group and the behavioral education–alone group. The changes in CARS were −7.9 ± 5.42 for combination therapy and −4.6 ± 5.53 for behavioral education alone. For the purpose of calculating the standard deviation of the CARS change, the before-after correlation coefficient was assumed to be 0.33, referring to Sun *et al*. (2016) [33]. The sample size was calculated by using a two-tailed 5% significance level, 80% power, and 1:1 allocation by PASS 14 (NCSS Statistical Software, Kaysville, UT, USA). The alternative hypothesis was established that the change in CARS for 12 weeks between the experimental group and the control group was not the same. The required number of subjects per group is 45, and the total number of enrolled subjects is 120 (60 in each group), allowing for a 25% withdrawal rate.

### Statistical analysis

Data coding will be kept by the researcher team, and data analysis will be conducted by statisticians who are independent of the research team. Effectiveness data will be analyzed mainly with full analysis set (FAS) and further analyzed with per protocol set (PPS). The FAS is defined as all subjects who have taken at least one prescription in the clinical trial and have at least one measurement of the primary outcome after having the prescription. The PPS is defined as all subjects who have taken more than 118 packs (70% of the total 168 packs). The safety analysis includes all data from the participants who have taken at least one prescription in the clinical trial.

Continuous data will be presented as descriptive statistics such as mean and standard deviation, and any statistical differences between the two groups will be tested by using the independent *t* test or Wilcoxon rank-sum test. Categorical data will be presented as frequency and percentage, and any statistical differences between the two groups will be tested by using the chi-squared test or Fisher’s exact test. If significant differences of baseline are recognized between two groups, analysis of covariance (ANCOVA) or rank ANCOVA will be used. In secondary analyses within groups, continuous data will be analyzed by using the paired *t* test or Wilcoxon signed-rank test, and categorical data will be analyzed by using McNemar’s test.

The significance level is 0.05 for all statistical tests, and two-tailed tests will be performed. Missing values will be imputed by the last-observation-carried-forward method.

### Data monitoring

Censors of K-CTC will visit each hospital to monitor protocol violations, recruitment rate, document reporting, participant compliance, and AEs during the trial. Visit procedure and schedule will follow the standard operating procedure of K-CTC. The detected items shall be properly discussed with the principal investigator and the researchers in each hospital.

### Ethics and dissemination

The IRB of two hospitals will review the protocol at least annually. Any modifications to the protocol will be agreed to by the principal investigator and approved by each IRB prior to implementation. Korean medicine doctors will introduce the trial to guardians and obtain written informed consent. If AEs occur during the trial, appropriate medical care will be provided until the subject recovers. All participant data will be identified by the code number and participants’ initials, and the information will be stored in locked file cabinets with limited access. Investigators will have direct access to their own hospital’s data set, and the principal investigator will be given access to full data sets. Unidentified data will be shared by request within 3 years after the end of trial. The study results will be released to participants, health-care professionals, and the public via publications. The authors of those publications will participate in this trial for longer than 1 year.

## Discussion

Herbal medicines which have been shown to be effective for ASD in RCTs include Jiawei Wendan decoction, modified Yinhuo decoction, and supplemented Lizhong decoction [34]. Modified Yinhuo decoction and supplemented Lizhong decoction improve CARS and the total effective rate, and Jiawei Wendan decoction improves the total effective rate. However, there were some methodological limitations. For example, the number of participants in each group was less than 30 and all studies were unblinded. Our study will be the first prospective, multicenter, randomized, double-blinded, placebo-controlled, parallel-group clinical trial assessing the safety, effectiveness, and economics of an herbal medicine combined to acupuncture in the treatment of ASD.

ASD is generally diagnosed around 4 years of age [35], and the UGSJB dose administered to 4- to 6-year-old children ages is half that of adults. Moreover, the golden time for ASD treatment is known to be between the ages of 2 and 6 [36]. We decided to enroll 4- to 6-year-old children with ASD for the best effectiveness using a single capacity of UGSJB. The treatment period was set to be 3 months, following the previously effective herbal treatment RCTs. Acupuncture, a treatment supported by the National Health Service in South Korea, will be implemented in all groups for economic comparison. The behavior of children with ASD will be evaluated by CARS, ABC^1^, and ABC^2^. Social maturity will be assessed by SMS. Quality of life will be evaluated by CHQ-PF28 and EQ-5D-Y proxy 1. Stress of parents of children with ASD will be assessed by K-PSI-SF. The safety of USGJB will be investigated on the basis of voluntarily reported AEs, and the economic evaluation of USGJB will be analyzed by CEA, CUA, and CBA.

There were a few concerns about planning the study protocol. First, there are no limitations to concurrent treatment except for risperidone and aripiprazole. Therefore, it may be difficult to manage all interventions because of the wide variation in each participant’s concurrent treatment. However, there is no standard treatment for ASD, and ASD is often treated case by case because of the broad spectrum and individual differences in symptoms. Therefore, we decided to verify the effects of herbal medicine added to acupuncture while maintaining existing treatments throughout the clinical trial, which is similar to the management protocols of clinical Korean medicine. Second, it was discussed that the subjectivity of evaluators could affect overall outcomes since all outcome variables will be obtained through questionnaires. In order to reduce this bias, we decided to use placebo to keep blinding strictly from researchers and evaluators, unlike other existing RCTs of herbal medicines on ASD. Third, owing to the intractable nature of disease, changes in symptoms may not be noticeable in the short period of 3 months. Therefore, we decided to reassess the symptoms after 3 months when the interventions are completed.

UGSJB treats aggressive behavior by suppressing excess glutamateric neuron activity in the hippocampus [37], anxiety through the serotonergic neurotransmission pathway [38], chronic pruritus by inhibiting nerve growth factor–induced neurite growth [39], and insomnia by increasing stage 2 sleep and decreasing sleep latency [40]. UGSJB has some neuronal effects and was reported to improve symptoms of ASD in a case report [41]. However, these effects have not been demonstrated in a clinical trial. Ukgansan, which is a variant of UGSJB, has shown only some experimental and anecdotal improvement of ASD symptoms. Our RCT will provide results on the safety, effectiveness, and economic value of UGSJB for ASD, and treatment options with higher evidence may be provided to children with ASD.

### Trial status

Protocol version number and date: version 2.3 (June 1, 2018). Date recruitment began: July 1, 2018. Approximate date when recruitment will be completed: Dec. 31, 2020.

### Additional file

Additional file 1: SPIRIT (Standard Protocol Items: Recommendations for Interventional Trials) 2013 Checklist: Recommended items to address in a clinical trial protocol and related documents*. (DOC 122 kb)

## Data Availability

The datasets used or analyzed (or both) during the current study are available from the corresponding author on reasonable request.

## A. Appendix

**Table 2:** Protocol versions

Version	Date	Action
1.0	2017-Jul-06	Protocol draft development
1.1	2017-Jul-13	Statistical review
1.2	2017-Aug-02	Ethical issue review
1.3	2017-Aug-04	Deleting EHWA-Checklist for Autistic Children as an outcome measure
1.7	2017-Dec-06	Revision following institutional review board’s comments
2.0	2018-Jan-10	Revision of inclusion criteria and Aberrant Behavior Checklist
2.1	2018-Mar-13	Amendment of Investigational New Drug review
2.2	2018-Mar-22	Additional amendment
2.3	2018-Jun-01	Minor amendment including typing errors

## Abbreviations

ABC^1^: Autism Behavior Checklist
ABC^2^: Aberrant Behavior Checklist
ACER: Average cost-effectiveness ratio
ACUR: Average cost-utility ratio
AE: Adverse event
ANCOVA: Analysis of covariance
ASD: Autism spectrum disorder
CARS: Childhood Autism Rating Scale
CBA: Cost-benefit analysis
CEA: Cost-effectiveness analysis
CHQ-PF28: Child Health Questionnaire-Parent Report Form 28
CUA: Cost-utility analysis
EQ-5D-Y: EuroQoL Five-dimension Five-level Youth
FAS: Full analysis set
ICER: Incremental cost-effectiveness ratio
IRB: Institutional review board
K-CTC: Korean Medicine Clinical Trial Center
K-PSI-SF: Korean Parenting Stress Index Short Form
PDD: Pervasive developmental disorder
PPS: Per protocol set
PSI: Parenting Stress Index
RCT: Randomized controlled trial
SMS: Social Maturity Scale
TCM: Traditional Chinese medicine
UGSJB: Ukgansangajinpibanha
